# Supplementary material for: Real-time imaging of rotation during synthesis by the replisome
Source: bioRxiv. 2025 Apr 7:2025.04.01.646591. Preprint. [Version 1] doi: 10.1101/2025.04.01.646591 (PMC12026505; doi:10.1101/2025.04.01.646591)
Supplement: Supplement 3 [file NIHPP2025.04.01.646591v1-supplement-3.pdf]

bioRxiv preprint doi: https://doi.org/10.1101/2025.04.01.646591; this version posted April 7, 2025. The copyright holder for this preprint (which was not certified by peer review) is the author/funder, who has granted bioRxiv a license to display the preprint in perpetuity. It is made available under aCC-BY 4.0 International license.

Supplementary Information

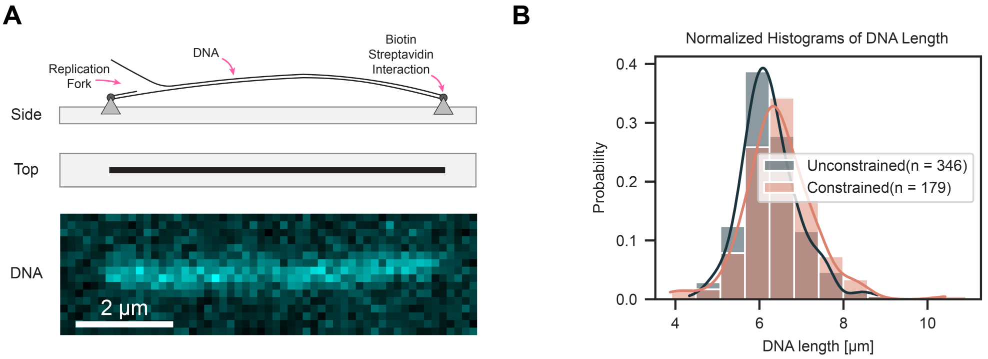

Figure S1. Molecule length distributions.
(**A**) DNA attachment in linear flow cell configuration. Cartoon indicates replication fork where replication is initiated. DNA is tethered to the surface utilizing a biotin streptavidin interaction. (**B**) Length Distribution for the two substrate types in µm. Number of molecules is indicated in the legend. Unconstrained and constrained are stretched 68±7% and 69±9% (Mean ± SD), respectively.

bioRxiv preprint doi: https://doi.org/10.1101/2025.04.01.646591; this version posted April 7, 2025. The copyright holder for this preprint (which was not certified by peer review) is the author/funder, who has granted bioRxiv a license to display the preprint in perpetuity. It is made available under aCC-BY 4.0 International license.

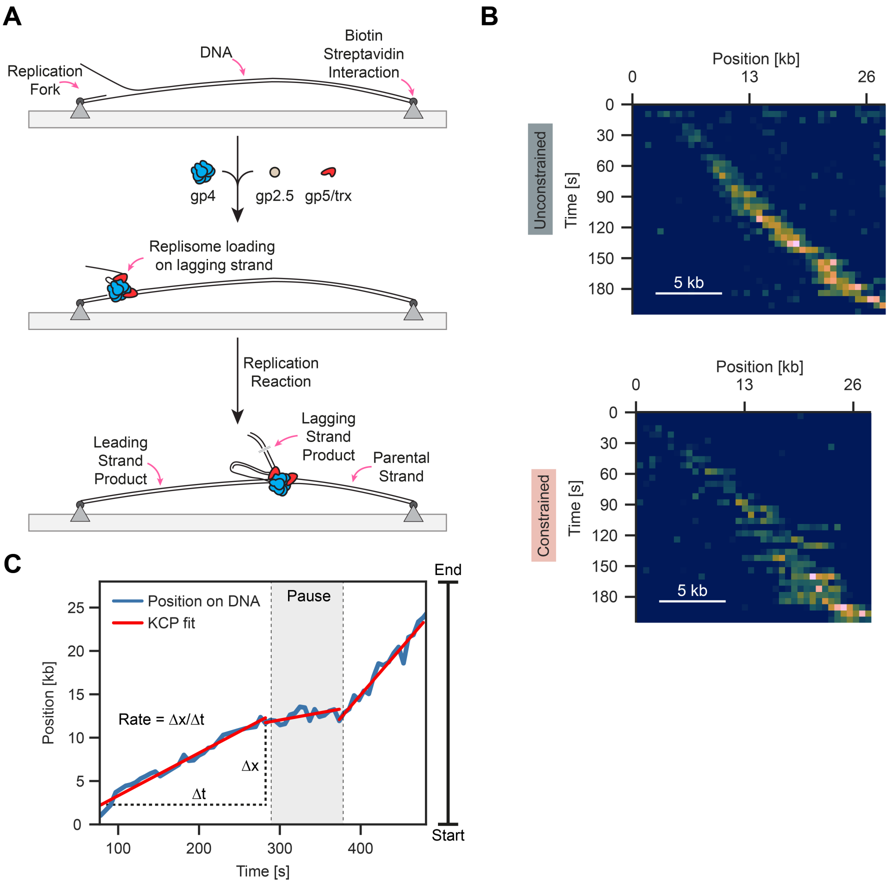

Figure S2. Replication using a linear flow configuration.
(**A**) Schematic of replication. DNA is tethered to the surface using biotin-streptavidin interactions. T7 replication components initiate replication on the preformed fork. Leading strand product forms behind the replisome while the lagging strand product forms a blob at the location of the replisome used to track progression. (**B**) Example kymographs for single molecule replication on unconstrained and constrained molecules. (**C**) Single-molecule tracking of lagging-strand product resulting in a time trace containing replication rates and processivities.

bioRxiv preprint doi: https://doi.org/10.1101/2025.04.01.646591; this version posted April 7, 2025. The copyright holder for this preprint (which was not certified by peer review) is the author/funder, who has granted bioRxiv a license to display the preprint in perpetuity. It is made available under aCC-BY 4.0 International license.

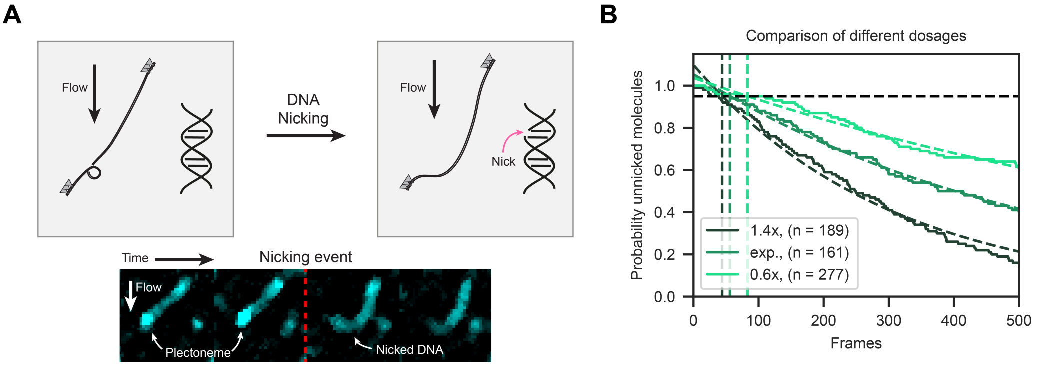

Figure S3. Nick detection assay for quantification of laser-induced damage.
(**A**) Concept of nicking assay to control for imaging condition introducing nick in individual strands. DNA is supercoiled and upon nick introduction supercoils are relaxed and result in an increase in the overall size of the DNA shape. (**B**)Survival curve for unnicked molecules over time fit with an exponential decay curve. Three different laser settings were compared. 1.4 times and 0.6 times the laser setting of the experimental condition ('exp') are displayed.

bioRxiv preprint doi: https://doi.org/10.1101/2025.04.01.646591; this version posted April 7, 2025. The copyright holder for this preprint (which was not certified by peer review) is the author/funder, who has granted bioRxiv a license to display the preprint in perpetuity. It is made available under aCC-BY 4.0 International license.

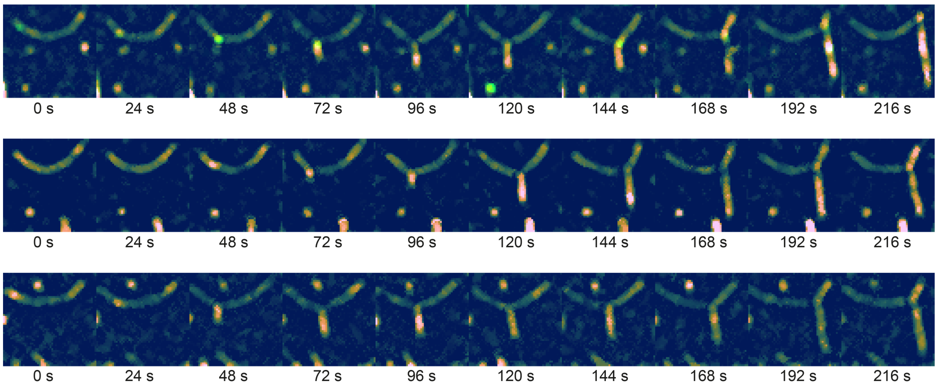

Figure S4. Representative molecules for transverse flow imaging of DNA replication (Unconstrained). Three representative kymographs for replication reaction in transverse flow using unconstrained substrate.

bioRxiv preprint doi: https://doi.org/10.1101/2025.04.01.646591; this version posted April 7, 2025. The copyright holder for this preprint (which was not certified by peer review) is the author/funder, who has granted bioRxiv a license to display the preprint in perpetuity. It is made available under aCC-BY 4.0 International license.

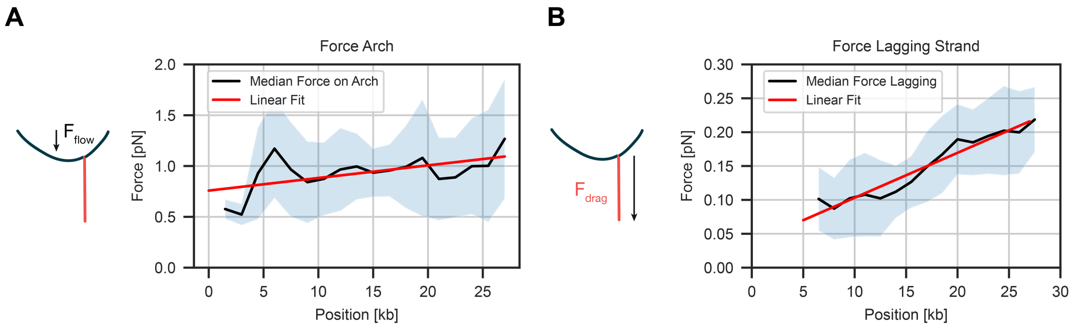

Figure S5. Estimation of applied forces from transverse flow.
(**A**) Force on the arch applied by the flow. Force was calculated comparing arch extension to the contour length of DNA substrate. (**B**) Applied force on the lagging-strand product due to transverse flow as a function of product position. Worm-like chain model was used for force estimation and a linear fit was added. Standard error of the mean indicates error margin.

bioRxiv preprint doi: https://doi.org/10.1101/2025.04.01.646591; this version posted April 7, 2025. The copyright holder for this preprint (which was not certified by peer review) is the author/funder, who has granted bioRxiv a license to display the preprint in perpetuity. It is made available under aCC-BY 4.0 International license.

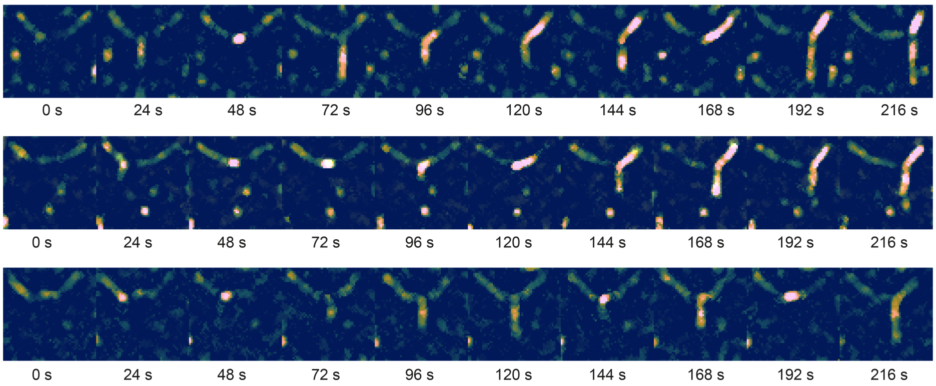

Figure S6. Representative molecules for transverse flow imaging of DNA replication (Constrained).
Three representative kymographs for replication reaction in transverse flow using constrained substrate.

bioRxiv preprint doi: https://doi.org/10.1101/2025.04.01.646591; this version posted April 7, 2025. The copyright holder for this preprint (which was not certified by peer review) is the author/funder, who has granted bioRxiv a license to display the preprint in perpetuity. It is made available under aCC-BY 4.0 International license.

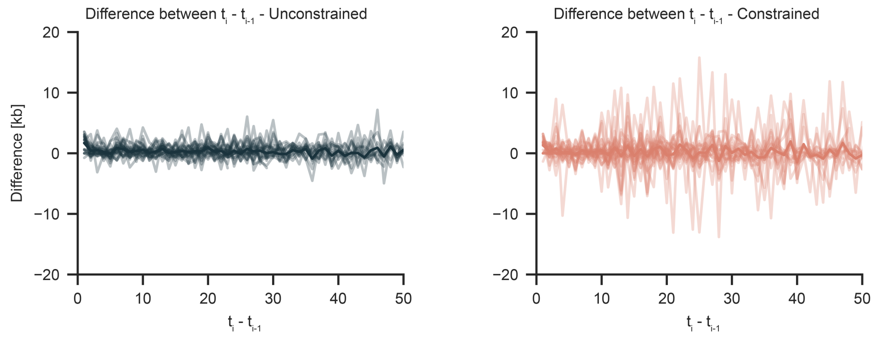

Figure S7. Transformed lagging strand length.
Transformation of lagging strand length over time (Fig. 4F) from non-stationary function to stationary function. For transformation the difference is taken for each point by subtracting the value from the previous timepoint.

bioRxiv preprint doi: https://doi.org/10.1101/2025.04.01.646591; this version posted April 7, 2025. The copyright holder for this preprint (which was not certified by peer review) is the author/funder, who has granted bioRxiv a license to display the preprint in perpetuity. It is made available under aCC-BY 4.0 International license.

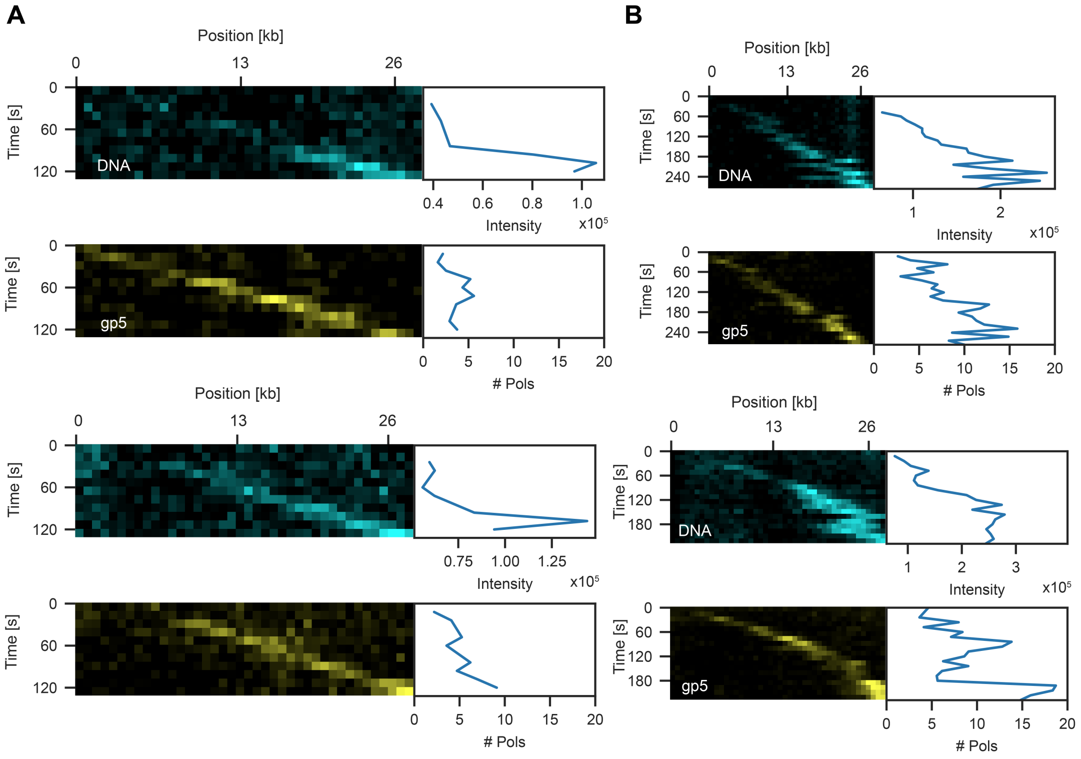

Figure S8. Representative molecules for labelled polymerases during DNA replication.
(**A**) DNA replication events in the absence of flow on unconstrained molecules. (**B**) DNA replication events in the absence of flow on constrained molecules. Stained DNA is displayed in cyan on the top and polymerase signal is displayed in yellow on the bottom.

bioRxiv preprint doi: https://doi.org/10.1101/2025.04.01.646591; this version posted April 7, 2025. The copyright holder for this preprint (which was not certified by peer review) is the author/funder, who has granted bioRxiv a license to display the preprint in perpetuity. It is made available under aCC-BY 4.0 International license.

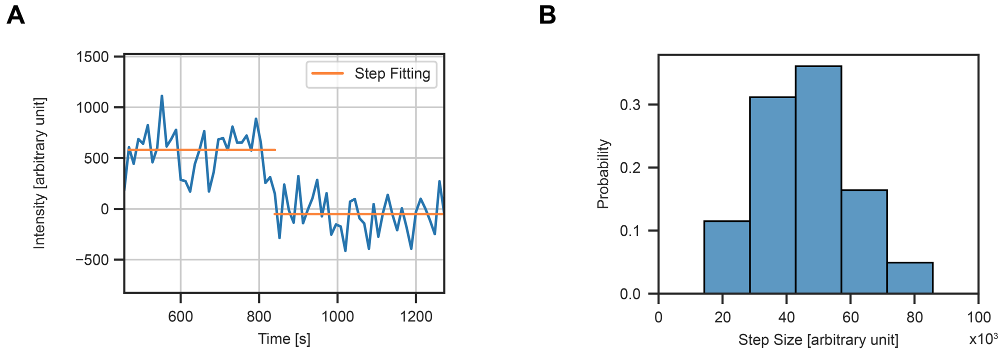

Figure S9. Estimating fluorescent signal of a single labelled polymerase.
(**A**) Bleach step of a surface immobilized polymerase. Step was fitted using change point analysis. (**B**) Distribution of different step sizes for one single molecule dataset.

bioRxiv preprint doi: https://doi.org/10.1101/2025.04.01.646591; this version posted April 7, 2025. The copyright holder for this preprint (which was not certified by peer review) is the author/funder, who has granted bioRxiv a license to display the preprint in perpetuity. It is made available under aCC-BY 4.0 International license.

Supplementary Videos

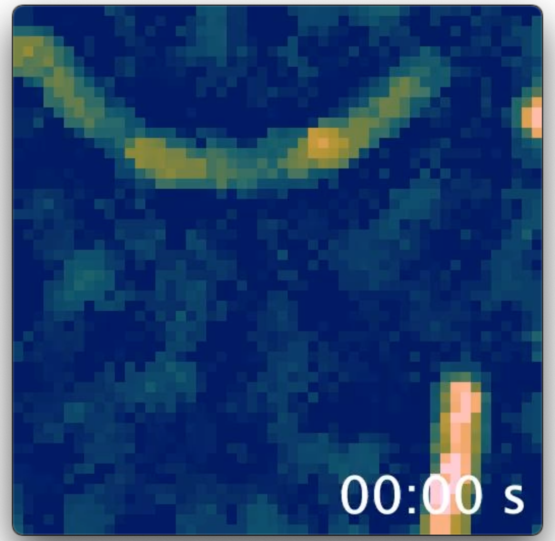

Figure Movie S1.
Representative unconstrained molecule during DNA replication imaged using transverse flow. The individual leading, lagging, and parental strands are all spatially resolved. The lagging-strand product is seen growing out from along the arch and extending downward due to applied flow. Intensity is color-coded using the Batlow LUT. Time is displayed as minutes and seconds in the format (mm:ss).

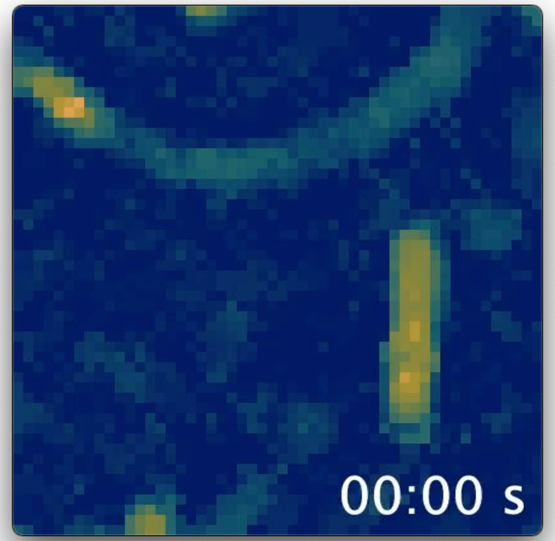

Figure Movie S2.
Representative constrained molecule during DNA replication imaged using transverse flow. The lagging-strand product is seen wrapping around the arch during ongoing DNA synthesis due to fork rotation. Transient downward extension events are observed in between bursts of fork rotation. Intensity is color-coded using the Batlow LUT. Time is displayed as minutes and seconds in the format (mm:ss).

bioRxiv preprint doi: https://doi.org/10.1101/2025.04.01.646591; this version posted April 7, 2025. The copyright holder for this preprint (which was not certified by peer review) is the author/funder, who has granted bioRxiv a license to display the preprint in perpetuity. It is made available under aCC-BY 4.0 International license.

Supplementary Tables

Experimental Type	DNA Type	Mean Burst Rate [bp/s] Mean±SEM	Processivity [kb] Mean±SEM	Fraction of Fully replicated molecules [%]	Number of molecules
Linear	Unconstrained	99 ± 3	24.6 ± 0.3	75	186
	Constrained	96 ± 5	23.6 ± 0.6	57	79
Transverse	Unconstrained (leading)	97 ± 7	25.0 ± 0.8	67	24
	Constrained (leading)	98 ± 9	19.3 ± 1.4	32	26
Pausing					
Experimental Type	DNA Type	0 Pause	1 Pause	2 Pause	Number of molecules
Linear	Unconstrained	0.88	0.12	0.01	186
	Constrained	0.78	0.18	0.04	79
Blob size					
Experimental Type	DNA Type	Blob size [kb] Mean±SD			Number of molecules
Linear	Unconstrained	3.3 ± 0.7			114
	Constrained	4.4 ± 1.3			52
Number of Polymerases					
Experimental Type	DNA Type	Mean number of polymerases Mean±SEM			Number of molecules
Linear	Unconstrained	3.8 ± 0.1			75
	Constrained	11.4 ± 0.2			56

Table S1. Summary statistics from single-molecule microscopy experiments organized by experimental conditions reporting rates, processivities, fraction fully replicated and molecule numbers.

Name	Sequence
Oligo1	TATTAGCGGCCGCGATTGTTCTTTATTCATTTT
Oligo2	CCCATCGGAAAACTCCTGCTTTAGC
Oligo3	TTACCGCATACCAATAACGCTTCAC
Oligo4	GAAACTCAACATCGTCATCAAACGC
Oligo5	[BIO]AGGTCGCCGCCC
Oligo6	[PHO]TCGAGGGCGGCGACCT
Oligo7	[PHO]CTAGAGACAGCAAGTTGGACAATCCATCTCGTTCTATCACTAATGCAGGGAGGATTTCAGATATGGCAACTAGTATGCCG
Oligo8	TTTTTTTTTTTTTTTTTTTTTTTTTTTTTTTATGGATTGTCCAACTTGCTGTCT
Oligo9	[BIOTEG]TTTTTTTTTTCGGCATACTAGTTGCCATATCTGAAATCCTCCCTGC

Table S2. List of sequences used to create replication substrates for single-molecule experiments.
